# Synergistic combinatorial antihyperlipidemic study of selected natural antioxidants; modulatory effects on lipid profile and endogenous antioxidants

**DOI:** 10.1186/s12944-016-0323-3

**Published:** 2016-09-09

**Authors:** Peer Abdul Hannan, Jamshaid Ali Khan, Irfan Ullah, Safi Ullah

**Affiliations:** Department of Pharmacy, University of Peshawar, Peshawar, 25120 Khyber Pakhtunkhwa Pakistan

**Keywords:** Hyperlipidemia, Atherosclerosis, Redox homeostasis, Synergism, Lipid peroxidation

## Abstract

**Background:**

Hyperlipidemia, a major pathological condition associated with disrupted lipid levels and physiological redox homeostasis. The excessive release of reactive oxygen species (ROS) leads to enhanced lipid peroxidation, aggravated atherosclerosis and oxidative stress. Integration of natural antioxidant blends in alone or with conventional treatments can alleviate these issues synergistically contributing least side effects. Published literature reported the efficacy of natural antioxidants as individual and in combinations in various conditions but less data is available on their evaluation in low dose ratio blends particularly in hypercholesterolemic diet.

**Methods:**

Antihyperlipidemic effects of selected natural antioxidants; the phenolic oligomeric proanthocyanidins (OPC) and pterostilbene (PT) with niacin (NA) were investigated in current study. Their effects on lipid profile, lipid peroxidation and their aptitude to establish redox state between oxidants and antioxidants in body were evaluated in high cholesterol diet fed animal model. Male albino rabbits (*n* = 6) weighing 1.2–1.6 kg, supplemented with high cholesterol diet (400 mg/kg) for 12 weeks were used in the experiment. Antioxidants were administered individual high (100 mg/kg) and in low dose combinations (total dose = 100 mg/kg). Student’s *t* test and one way analysis of variance (ANOVA) followed by Dunnet’s test were used as statistical tools for evaluation.

**Results:**

The results showed synergistic effects of low dose antioxidant blends. Therapies retarded elevation in blood lipid levels, lipid peroxidation and blood antioxidant depletion and consequently contributed in reestablishing redox homeostasis. The LDL/HDL ratio and atherogenic index were suppressed significantly in blend therapies with maximum effects of 59.3 and 25 % (*p* >0.001) observed in 50:30:20 ratios of OPC, NA and PT, compared to individual therapies 37 and 18 % max respectively. Moreover the results were also in close proximity with the statin therapy (52.66, 26.28 %).

**Conclusion:**

This study provides an evidence for natural antioxidants blends superiority over individual therapy in chronic diseases like hyperlipidemia. Such therapies in human equivalent doses can help in mitigating chronic illnesses in general populations.

## Background

Hyperlipidemia is a major pathological condition associated with mortality and disability in both developed and developing countries [1]. Chronic diseases like obesity, diabetes, atherosclerosis, coronary heart disease are closely interconnected to hyperlipidemia. Intake of high fat diet and lack of exercise are the major causes of hyperlipidemia. In normal metabolic processes; reactive oxygen species (ROS) are released as byproducts. They act not only as essential part of immunity, in signaling transduction and other vital processes but also responsible for detrimental effects through peroxidation of lipid bilayers. Therefore to maintain the physiological redox environment, the excess radicals are get scavenged by blood antioxidants [2]. Hyperlipidemia disrupts this redox balance and cause abnormal and uncontrolled release of free radicals resulting in accelerated atherogenic events developing a state of oxidative stress [3]. Endothelial cell membrane and unsaturated lipids including *trans* fatty acids is the main content of circulating low density lipoproteins (LDL) are the most vulnerable sites for free radical attack. Their oxidation leads to severe endothelial cell injury and atherosclerosis [4–6]. The saturated fats do not undergo lipid peroxidation therefore has no direct effect on oxidative stress biomarkers [7]. However when esterified with poly unsaturated fatty acids (PUFA), which is the readily oxidizable content, the esters gets oxidized initiating the inflammation and atherogenesis [8]. The antioxidants present in blo od and organs including glutathione, ascorbic acid, α-tocopherol fights against the progression of oxidative stress to regain the redox homeostasis but gets depleted in chronic conditions like hyperlipidemia [9, 10]. In such circumstances interventions with natural antioxidants supplements were found effective in normalizing lipid profile and blood antioxidant levels as reported previously [4, 11, 12]. Research data is well documented and supported the notion regarding their efficacies in chronic pathologies such as hyperlipidemia [13–16].

Natural antioxidants were evaluated in individual and in combinations for lipid lowering and free radical scavenging properties and were found effective in depressing the disease progression [14–22]. In current study natural antioxidants including oligomeric proanthocyanidins (hydrophilic), pterostilbene (lipophilic) and niacin were evaluated for extent of synergism by observing their effects in both individual and blended therapies.

Oligomeric proanthocyanidins (OPC) is a polyphenolic compound belongs to flavonoids class, widely distributed in plants and posses potent antioxidant [23] and lipid modulatory effects [24, 25]. Pterostilbene (PT) a phytoalexin, analogue of resvaretrol belong to the class stilbenes, found in grapes majorly and possess antihyperlipidemic, antidiabetic, antioxidant properties [26]. It is also a potent activator of fatty acid metabolism [27]. Niacin or nicotinic acid (NA) found in natural products and well known for its antihyperlipidemic and antioxidant activities [28–30]. To best of our knowledge less data was extracted about lipid lowering effect of pterostilbene with OPC and NA in blend therapies especially low doses in orally fed high cholesterol diet models. The current study was designed to investigate the extent of synergism of selected hydrophilic/lipophilic natural antioxidants in assorted combination therapies in “hyperlipidemic animal model” using rabbits. Antioxidants were administered in individual and in low dose combination ratios. Atorvastatin was used as a standard for comparative analysis. Effects were evaluated against negative control group kept on cholesterol only. Serum lipid profiles were analyzed at predetermined intervals using standard kit method, while blood antioxidants and lipid peroxidation were evaluated using HPLC methods [31, 32].

## Results and discussions

Acute rise in lipid profiles were observed in rabbits administered with cholesterol for 2 weeks compared to healthy group. Almost two to four time rise was observed in every parameter. Student’s *t* test was applied to evaluate the difference statistically. Lipid profiles are calculated as mean ± SEM (*n* = 6) along with *p*-values in Table 1.

**Table 1:** Lipid profiles of healthy and hypercholesterolemic animals

Parameter	Healthy rabbits	After 2 weeks cholesterol dosing	*p*-value
	Mean ± SEM (*n* = 6)	Mean ± SEM (*n* = 6)	
Total cholesterol (mg/dl)	61 ± 2.468	249 ± 7.425	<0.0001
Triglycerides (mg/dl)	67 ± 0.635	140 ± 2.465	<0.0001
LDL-Cholesterol (mg/dl)	16.36 ± 0.618	172.3 ± 5.557	<0.0001
HDL-Cholesterol (mg/dl)	31.24 ± 1.241	48.72 ± 2.211	<0.0001
VLDL-Cholesterol (mg/dl)	30.45 ± 1.002	63.64 ± 2.113	<0.0001
LDL/HDL ratio	0.524 ± 0.035	3.536 ± 0.040	<0.0001
Atherogenic Index (AI)	0.331 (0.014)	0.458 (0.040)	0.0071

*p*-values acquired using student’s *t* test between columns

*SEM* standard error of mean

### Effect of exogenous antioxidant therapy on serum lipid profile

Single drug therapies showed significant effects in reducing lipid levels, confirmed by LDL/HDL ratio and atherogenic index (AI). The OPC, NA and PT maintained LDL/HDL ratios by 19.49 ± 0.87 (*p* <0.001), 20.26 ± 0.94 (*p* <0.001) and 19.19 ± 0.91 (*p* <0.01) and AI values 0.893 ± 0.033 (*p* <0.01), 0.896 ± 0.053 (*p* <0.01), 0.971 ± 0.073 (*p* <0.05) respectively. The atorvastatin therapy showed 14.0 ± 0.99 (*p* <0.001) and 0.785 ± 0.061 (*p* <0.001) compared to disease control 30.50 ± 0.69, 1.096 ± 0.040. In combination therapies the results were even more significant and synergism were observed by improving blood lipid profile depending on dose ratio in particular combination. Among combinations of OPC with NA the 70:30 ratio delivered significant effects by suppressing LDL/HDL ratio to 15.36 ± 0.98 (*p* <0.001) while differ nonsignificantly from other two ratios 16.77 ± 0.69 (*p* <0.001) and 17.33 ± 0.75 (*p* <0.001). AI was significantly reduced to 0.843 ± 0.041 (*p* <0.001), 0.837 ± 0.045 (*p* <0.001) and 0.887 ± 0.037 (*p* <0.01) in 70:30, 30:70 and 50:50 respectively. Similarly in OPC with PT combinations all the ratios provided significant effects compared to disease control but no significant differences were observed between the groups. The LDL/HDL and AI were 14.60 ± 0.98 (*p* <0.001) and 0.851 ± 0.041 (*p* <0.001) in 70:30 ratio, 15.19 ± 0.42 (*p* <0.001), 0.929 ± 0.057 (*p* <0.05) in 30:70 and 16.33 ± 0.75 (*p* <0.001), 0.906 ± 0.037 (*p* <0.01) in 50:50 respectively. In NA and PT, the 70:30 and 50:50 combinations induced similar effects on LDL/HDL ratios 15.16 ± 0.89 and 15.16 ± 0.84 (*p* <0.001) respectively, while comparing AI values results were more profound in 70:30 (0.858 ± 0.053) (*p* <0.001) compared to other groups 0.919 ± 0.037 (*p* <0.05) and 0.868 ± 0.037 (*p* <0.01) respectively. In general the two drug combinations showed synergism, evident from significant outcome however differ less from one another [33].

The ratios with optimum lipid lowering effects in above therapies were subsequently molded in to three drug combinations to evaluate the extent of synergism of antioxidants acting through various mechanisms. The antioxidant with highest ratio (70 %) was reduced by 20 mg and this amount was replaced with third candidate. All three drug combinations showed more significant results as compared to two drug combinations. Most significant effects were observed in 50:30:20 ratio of OPC, NA and PT which suppressed LDL/HDL ratio by 59.3 % and AI by 25 % (*p* <0.001) comparable with the statin outcome 52.66, 26.28 % (*p* <0.001) respectively. Though there were minor differences among the groups but differ significantly from the disease control. The high dose single drug therapies suppressed LDL/HDL by 37 % and AI by 18.5 % maximum, which are much less as compared to low dose blend therapies confirmed the superiority of combinational therapies. Lipid profiles in mean ± SEM and %change in blood lipids at 95 % confidence interval are summarized in Tables 2 and 3.

**Table 2:** Blood lipid profile of various groups after 12 weeks of study duration

Parameters								
GROUP		TC value (mg dl^−1^) ± SEM	LDL-C value (mg dl^−1^) ± SEM	HDL-C value (mg dl^−1^) ± SEM	TG value (mg dl^−1^) ± SEM	VLDL-C (mg dl^−1^) Value ± SEM	LDL/HDL ratio ± SEM	Atherogenic index ± SEM
1	Blank Control	105 ± 5.013	58.75 ± 18.25	30.65 ± 1.74	78 ± 3.527	35.45 ± 3.074	1.917 ± 0.56	0.406 ± 0.037
2	Disease Control	946 ± 38.53	848.8 ± 34.19	27.83 ± 0.82	347 ± 10.04	157.7 ± 2.29	30.50 ± 0.69	1.096 ± 0.040
3	Atorvastatin	*** 625 ± 17.42	*** 539.65 ± 15.01	*** 37.35 ± 1.72	*** 240 ± 5.49	*** 109.1 ± 3.35	*** 14.45 ± 0.99	*** 0.808 ± 0.061
4	Proanthocyanidins (OPC)	*** 733 ± 21.42	*** 647.8 ± 19.34	ns 33.24 ± 2.09	*** 260 ± 9.10	*** 118.2 ± 2.09	*** 19.49 ± 0.87	** 0.893 ± 0.033
5	Nicotinic acid (NA)	** 806 ± 16.51	** 715.1 ± 19.87	* 35.29 ± 1.85	** 278 ± 8.66	** 126.4 ± 4.79	*** 20.26 ± 0.94	** 0.896 ± 0.053
6	Pterostilbene (PT)	*** 698 ± 14.19	*** 607.16 ± 14.45	ns 31.64 ± 3.03	* 296 ± 3.64	* 134.5 ± 6.30	*** 19.19 ± 0.91	* 0.971 ± 0.073
7	OPC : NA (30:70)	*** 725 ± 15.92	*** 635.1 ± 14.62	*** 37.86 ± 1.88	*** 260 ± 6.26	*** 118.2 ± 3.93	*** 16.77 ± 0.69	*** 0.837 ± 0.045
8	OPC : NA (50:50)	*** 704 ± 13.15	*** 614.0 ± 12.58	* 35.42 ± 1.36	*** 273 ± 8.68	** 124.1 ± 3.76	*** 17.33 ± 0.75	** 0.887 ± 0.037
9	OPC : NA (70:30)	*** 642 ± 13.31	*** 555.4 ± 11.65	* 36.16 ± 2.13	*** 252 ± 8.54	*** 114.5 ± 3.38	*** 15.36 ± 0.98	*** 0.843 ± 0.041
10	OPC : PT (30:70)	*** 598 ± 14.01	*** 507.8 ± 10.38	ns 33.42 ± 1.68	** 284 ± 3.81	* 129.1 ± 4.17	*** 15.19 ± 0.42	* 0.929 ± 0.057
11	OPC : PT (50:50)	*** 642 ± 15.60	*** 553.5 ± 14.66	ns 33.89 ± 2.12	*** 273 ± 8.68	** 124.1 ± 3.76	*** 16.33 ± 0.75	** 0.906 ± 0.037
12	OPC : PT (70:30)	*** 608 ± 15.06	*** 518.0 ± 12.37	* 35.47 ± 2.16	*** 252 ± 8.95	*** 114.5 ± 3.38	*** 14.60 ± 0.98	** 0.851 ± 0.041
13	NA : PT (30:70)	*** 615 ± 15.02	*** 526.2 ± 9.57	ns 33.41 ± 1.61	*** 277 ± 11.1	** 125.9 ± 3.04	*** 15.75 ± 0.53	* 0.919 ± 0.037
14	NA : PT (50:50)	*** 646 ± 13.97	*** 555.4 ± 12.30	** 36.63 ± 1.54	*** 270 ± 7.07	** 122.7 ± 4.34	*** 15.16 ± 0.84	** 0.868 ± 0.037
15	NA : PT (70:30)	*** 659 ± 13.11	*** 567.6 ± 10.70	** 37.44 ± 2.26	*** 270 ± 5.91	** 122.7 ± 3.86	*** 15.16 ± 0.89	*** 0.858 ± 0.053
16	OPC : NA : PT (50:30:20)	*** 563 ± 13.31	*** 474.2 ± 11.55	*** 38.20 ± 1.89	*** 253 ± 6.65	*** 115.0 ± 3.04	*** 12.41 ± 0.72	*** 0.821 ± 0.041
17	OPC : NA : PT (50:20:30)	*** 572 ± 13.15	*** 482.67 ± 8.29	** 37.53 ± 1.34	*** 259 ± 9.46	** 120.5 ± 5.08	*** 12.86 ± 0.78	*** 0.839 ± 0.037
18	OPC : NA : PT (20:50:30)	*** 588 ± 11.73	*** 497.32 ± 13.06	*** 38.68 ± 2.37	*** 260 ± 2.81	*** 118.2 ± 2.83	*** 12.86 ± 1.17	** 0.827 ± 0.029

For a group of six animals, the lipid profiles were calculated as mean ± SEM. Treatment groups were compared with the disease control using ANOVA followed by Dunnet’s test (*p <0.05, ** *p* <0.01, ***p <0.001)

**Table 3:** Mean percent change in lipid profile after 12 weeks in each treatment group

Parameters (%)								
GROUP		TC value (95 % CI)	LDL-C value (95 % CI)	HDL-C value (95 % CI)	TG value (95 % CI)	VLDL-C (95 % CI)	LDL/HDL (95 % CI)	Atherogenic index (95 % CI)
1	Atorvastatin	−34.0 (−36.43, −31.57)	−36.42 (−39.00, −33.80)	34.20 (29.78, 38.62)	−30.80 (−32.74, −28.86)	−30.80 (−33.58, −28.02)	−52.66 (−62.56, −42.76)	−26.28 (−31.97, −20.59)
2	Proanthocyanidins (OPC)	−22.50 (−24.19, −20.81)	−23.68 (−25.50, −21.86)	19.44 (16.30, 22.58)	−25.07 (−27.33, −22.81)	−25.05 (−26.18, −23.92)	−36.09 (−40.21, −31.97)	−18.52 (−20.26, −16.78)
3	Nicotinic acid (NA)	−14.90 (−15.69, −14.11)	−15.75 (−16.87, −14.63)	26.8 (23.19, 30.41)	−19.88 (−21.48, −18.28)	−19.85 (−21.78, −17.92)	−33.57 (−37.57, −29.57)	−18.25 (−21.03, −15.47)
4	Pterostilbene (PT)	−26.22 (−27.59, −24.85)	−28.47 (−30.51, −26.43)	13.69 (10.32, 17.06)	−14.7 (−15.16, −14.24)	−14.71 (−16.48, −12.94)	−37.08 (−41.58, −32.58)	−11.4 (−13.12, −9.679)
5	OPC : NA 30:70	−23.36 (−24.68, −22.04)	−25.18 (−26.67, −23.69)	36.0 (31.41, 40.59)	−25.07 (−26.62, −23.52)	−25.05 (−27.19, −22.91)	−45.02 (−49.82, −40.22)	−23.63 (−26.88, −20.38)
6	OPC : NA 50:50	−25.58 (−26.81, −24.35)	−27.66 (−29.11, −26.21)	27.27 (24.54, 30.00)	−21.32 (−23.07, −19.57)	−21.31 (−22.97, −19.65)	−43.2 (−48.02, −38.38)	−19.07 (−21.10, −17.04)
7	OPC : NA 70:30	−32.13 (−33.84, −30.42)	−34.57 (−37.89, −31.25)	29.9 (25.39, 34.41)	−27.4 (−29.81, −24.99)	−27.4 (−29.48, −25.32)	−49.64 (−57.83, −41.45)	−23.08 (−25.94, −20.22)
8	OPC : PT 30:70	−36.79 (−39.00, −34.58)	−40.17 (−42.27, −38.07)	20.09 (17.50, 22.68)	−18.16 (−18.79, −17.53)	−18.13 (−19.63, −16.63)	−50.2 (−53.74, −46.66)	−15.24 (−17.65, −12.83)
9	OPC : PT 50:50	−32.13 (−34.13, −30.13)	−34.97 (−37.36, −32.58)	27.27 (22.98, 31.56)	−21.33 (−23.07, −19.59)	−21.31 (−22.97, −19.65)	−48.9 (−54.96, −42.84)	−19.07 (−21.11, −17.03)
10	OPC : PT 70:30	−35.73 (−38.01, −33.45)	−38.97 (−41.34, −36.60)	27.45 (23.16, 31.74)	−27.38 (−29.88, −24.88)	−27.39 (−29.47, −25.31)	−49.64 (−57.82, −41.46)	−22.35 (−25.11, −19.59)
11	NA : PT 30:70	−34.99 (−37.18, −32.80)	−38.0 (−39.77, −36.23)	20.05 (17.56, 17.56)	−20.17 (−22.24, −18.10)	−20.16 (−21.41, −18.91)	−48.36 (−52.55, −44.17)	−16.15 (−17.81, −14.49)
12	NA : PT 50:50	−31.71 (−33.47, −29.95)	−34.57 (−36.54, −32.60)	31.62 (28.20, 35.04)	−22.19 (−23.68, −20.70)	−22.19 (−24.20, −20.18)	−50.30 (−57.47, −43.13)	−20.8 (−23.07, −18.53)
13	NA : PT 70:30	−30.34 (−29.62, −26.82)	−33.13 (−32.21, −29.33)	34.53 (29.17, 39.89)	−22.2 (−23.45, −20.95)	−22.2 (−23.99, −20.41)	−50.30 (−55.64, −41.48)	−21.72 (−25.18, −18.26)
14	OPC : NA : PT 50:30:20	−40.49 (−42.95, −38.03)	−44.1 (−46.86, −41.34)	37.26 (32.61, 41.91)	−27.09 (−28.92, −25.26)	−27.08 (−28.92, −25.24)	−59.30 (−68.18, −50.42)	−25.09 (−28.30, −21.88)
15	OPC : NA : PT 50:20:30	−39.53 (−41.86, −37.20)	−43.13 (−45.10, −41.28)	32.5 (29.48, 35.52)	−25.36 (−27.52, −23.20)	−23.59 (−26.15, −21.03)	−57.84 (−66.56, −49.12)	−23.45 (−25.85, −21.05)
16	OPC : NA : PT 20:50:30	−37.80 (−39.74, −35.86)	−41.4 (−44.20, −38.60)	38.99 (32.84, 45.14)	−25.07 (−25.76, −24.38)	−25.05 (−26.59, −23.51)	−57.80 (−68.45, −47.15)	−24.50 (−26.67, −22.33)

Data is presented as mean percent change from the disease control at 95 % confidence interval

### Effect of exogenous antioxidant therapy on blood endogenous antioxidants

Effects of natural antioxidants therapies on blood antioxidants were evaluated by quantifying their levels in serum samples. They were quantified in normal, disease control, statin group, individual therapies groups, combination groups in each two drug ratios with optimum effects and all three drugs combinational therapies. Results showed initial rise in various antioxidants after 2 weeks of cholesterol dosing, presumably due to the over activation of antioxidant enzymes the catalase, glutathione peroxidase, superoxide dismutase and others; accelerating the production of GSH against peroxides radicals. Only NAC was found in reduced concentrations.

Significant up-regulation was observed in levels of methionine, GSH and NAC upto 35.08 ± 1.60, 3.82 ± 0.47 and 4.21 ± 0.425 μmol/L (*p* <0.001 each) respectively in combination therapies compared to disease control 19.30 ± 1.18, 3.01 ± 0.28 and 3.73 ± 0.412 μmol/L. Although in individual therapies the raised levels of methionine, GSH and NAC (24.73 ± 1.04 μmol/L (*p* <0.05), 3.38 ± 0.42 μmol/L (*p* <0.01) and 3.86 ± 0.404 μmol/L (*p* <0.05)) were less significant compared to combination therapies. The all-*trans* retinoic acid showed opposite results and increased parallel with the lipid levels from 1.80 ± 0.10 μmol/L (blank control) to 4.48 ± 0.24 μmol/L (disease control) while in treatment groups its elevation was retarded down to 3.26 ± 0.12 μmol/L (*p* <0.001) max; observed on 70:30 ratio of OPC and PT.

The improvement in GSH/GSSG ratios was significant in OPC (2.83 ± 0.065) (*p* <0.01) among individual therapies and more in combinational therapies ranging 2.51 ± 0.092 to 3.40 ± 0.097 (*p* <0.001), compared to disease control 2.12 ± 0.058. Homocysteine level was significantly controlled down to 10.09 ± 1.39 μmol/L (*p* <0.01), although nonsignificant variations were observed among treatment groups. Almost half of groups delivered nonsignificant effects on ascorbic acid levels with maximum effect of 5.29 ± 0.51 μmol/L (*p* <0.001) observed in 50:30:20 ratio of OPC, NA and PT compared to disease control 4.90 ± 0.59 μmol/L. The natural antioxidants were able to correct the antioxidant levels in blood and are more effective when used in blend therapies rather than individual as summarized in mean ± SEM in Tables 4 and 5.

**Table 4:** Quantification of water soluble endogenous antioxidants levels in various test groups

		Parameters (μmol/L)							
Groups		Cystine	Cysteine	Homo-cysteine	Methio-nine	Glutathione reduced	Glutathione oxidized	GSH/GSSG	N- acetyl cysteine
Baseline		52.95 ± 1.68	13.66 ± 0.87	8.72 ± 0.87	38.05 ± 1.55	3.75 ± 0.384	0.87 ± 0.037	4.42 ± 0.085	4.57 ± 0.457
After 2 weeks of CHO-dosing		93.35 ± 3.81	14.31 ± 1.09	9.08 ± 0.96	34.97 ± 1.77	4.13 ± 0.245	1.25 ± 0.041	3.26 ± 0.067	4.38 ± 0.441
*Observed changes after 90 days of treatment in groups*									
1	Blank control	60.37 ± 2.60	14.70 ± 1.49	8.56 ± 1.31	39.23 ± 1.88	3.78 ± 0.41	0.92 ± 0.029	4.18 ± 0.12	4.49 ± 0.429
2	Disease control	120.8 ± 6.41	42.36 ± 2.62	14.78 ± 1.49	19.30 ± 1.18	3.01 ± 0.28	1.44 ± 0.041	2.12 ± 0.058	3.73 ± 0.412
3	Atorvastatin	*** 79.59 ± 4.25	ns 36.70 ± 2.29	* 12.09 ± 1.63	ns 19.85 ± 1.26	** 3.38 ± 0.38	ns 1.35 ± 0.057	* 2.55 ± 0.081	* 3.83 ± 0.396
4	Proanthocyanidins (OPC)	*** 84.36 ± 3.39	ns 34.84 ± 2.13	* 11.44 ± 1.06	* 24.73 ± 1.04	** 3.38 ± 0.42	** 1.20 ± 0.049	** 2.83 ± 0.065	* 3.86 ± 0.404
5	Nicotinic acid (NA)	** 101.35 ± 3.89	ns 38.94 ± 1.95	* 12.85 ± 1.45	ns 20.51 ± 0.68	* 3.32 ± 0.41	* 1.24 ± 0.045	* 2.65 ± 0.090	ns 3.68 ± 0.416
6	Pterostilbene (PT)	** 98.81 ± 5.42	ns 36.75 ± 2.13	* 11.76 ± 1.12	ns 21.88 ± 1.16	ns 3.20 ± 0.42	ns 1.29 ± 0.041	* 2.51 ± 0.092	ns 3.80 ± 0.351
7	OPC: NA (70:30)	*** 87.23 ± 2.94	* 33.51 ± 1.41	** 10.87 ± 1.21	*** 29.83 ± 1.04	*** 3.60 ± 0.39	* 1.23 ± 0.045	** 2.90 ± 0.14	ns 3.77 ± 0.306
8	OPC: PT (70:30)	*** 83.77 ± 4.53	** 31.42 ± 2.37	* 11.34 ± 1.27	** 26.42 ± 0.87	*** 3.55 ± 0.45	** 1.19 ± 0.037	** 2.97 ± 0.082	** 3.95 ± 0.416
9	NA: PT (70:30)	*** 94.52 ± 3.39	** 31.88 ± 1.88	* 11.92 ± 1.06	*** 30.12 ± 1.32	*** 3.57 ± 0.36	ns 1.28 ± 0.053	* 2.78 ± 0.15	ns 3.74 ± 0.339
10	OPC: NA: PT (50:30:20)	*** 77.44 ± 3.87	*** 29.72 ± 1.46	** 10.09 ± 1.39	*** 35.08 ± 1.60	*** 3.82 ± 0.47	*** 1.12 ± 0.057	*** 3.40 ± 0.097	*** 4.14 ± 0.449
11	OPC: NA: PT (50:20:30)	*** 79.75 ± 3.89	*** 30.45 ± 1.59	** 10.64 ± 1.13	*** 28.58 ± 0.84	*** 3.77 ± 0.48	*** 1.17 ± 0.041	*** 3.25 ± 0.069	*** 4.21 ± 0.425
12	OPC: NA: PT (20:50:30)	*** 85.80 ± 3.05	*** 29.35 ± 1.93	** 11.17 ± 1.02	** 25.65 ± 1.23	*** 3.59 ± 0.45	*** 1.14 ± 0.049	** 3.15 ± 0.083	** 4.08 ± 0.400

For a group of six animals, the lipid profiles were calculated as mean ± SEM. Treatment groups were compared with the disease control using ANOVA followed by Dunnet’s test (*p <0.05, ** *p* <0.01, ***p <0.001)

**Table 5:** Quantification of fat soluble endogenous antioxidants, ascorbic acid and malondialdehyde levels in various test groups

Parameters (μmol/L)					
Group		α-Tocopherol	All-*trans* retinoic acid	Ascorbic acid	Malondialdehyde
Baseline		18.46 ± 1.74	1.83 ± 0.1143	5.59 ± 0.58	3.37 ± 0.233
After 2 weeks of CHO-dosing		17.29 ± 1.56	2.45 ± 0.1511	5.45 ± 0.67	4.08 ± 0.269
*Observed changes after 90 days of treatment in groups*					
1	Blank control	18.87 ± 1.49	1.80 ± 0.10	5.53 ± 0.72	3.49 ± 0.318
2	Disease control	11.30 ± 1.38	4.48 ± 0.24	4.90 ± 0.59	5.90 ± 0.404
3	Atorvastatin	* 12.57 ± 1.20	* 3.65 ± 0.22	ns 4.83 ± 0.49	*** 4.11 ± 0.376
4	Proanthocyanidins (OPC)	*** 14.79 ± 1.31	** 3.31 ± 0.21	* 5.06 ± 0.76	** 4.82 ± 0.425
5	Nicotinic acid (NA)	* 12.58 ± 0.98	ns 3.85 ± 0.25	ns 4.98 ± 0.49	* 5.01 ± 0.261
6	Pterostilbene (PT)	** 13.32 ± 1.03	** 3.44 ± 0.29	ns 5.01 ± 0.72	* 4.94 ± 0.396
7	OPC: NA (70:30)	*** 14.78 ± 1.67	* 3.53 ± 0.13	** 5.17 ± 0.76	** 4.66 ± 0.379
8	OPC: PT (70:30)	* 12.86 ± 1.47	*** 3.26 ± 0.12	** 5.09 ± 0.44	*** 4.35 ± 0.355
9	NA: PT (70:30)	** 13.28 ± 1.19	* 3.57 ± 0.17	ns 4.92 ± 0.46	** 4.75 ± 0.416
10	OPC: NA: PT (50:30:20)	*** 13.95 ± 1.89	** 3.29 ± 0.21	*** 5.29 ± 0.51	*** 4.14 ± 0.400
11	OPC: NA: PT (50:20:30)	* 12.76 ± 1.78	** 3.32 ± 0.16	** 5.21 ± 0.42	*** 3.96 ± 0.335
12	OPC: NA: PT (20:50:30)	*** 14.40 ± 1.75	* 3.54 ± 0.15	* 5.17 ± 0.48	*** 4.27 ± 0.412

For a group of six animals, the lipid profiles were calculated as mean ± SEM. Treatment groups were compared with the disease control using ANOVA followed by Dunnet’s test (*p <0.05, ** *p* <0.01, ***p <0.001)

### Discussions

Enhanced lipid peroxidation and endogenous antioxidant depletion are undoubtedly associated with hyperlipidemia [10, 34, 35]. This leads to a number of atherogenic effects including plaque formation in the vessel lumen and accelerating foam cells formation within vessel walls [36]. Natural antioxidants including those used in current study were applied in many chronic pathological conditions such as hyperlipidemia to prevent peroxidation, endogenous antioxidant depletion and suppression of oxidative stress progression [37, 38]. Natural antioxidants specially polyphenols present efficacy up to certain level [39, 40] above which they were found ineffective or as pro-oxidant by revoking their own beneficial effects. Similar effects were observed in ascorbic acid interventions, preventing pro-oxidant effects of LDL-associated α-tocopherol when gets oxidized in blood [41]. Same initiative was considered here that in place of using bulk dose individual therapies, the low dose combination therapies might provide superior results. Since each candidate deliver different antiperoxidative mechanisms preventing depletion of antioxidant cofactors. OPC act by up-regulating cytochrome (CYP) 7A1 enzyme induced bile secretion [24], lipid intestinal absorption inhibition and very low density lipoproteins (VLDL) secretion by liver [25]. PT work through up-regulating peroxisome proliferator-activated receptor (PPAR) [26]. NA by preventing lipid mobilization from tissues and consequent rise in plasma TG and free fatty acids (FFA) levels [29, 30, 42]. It also increases the plasma HDL levels and act as antioxidant when transformed into nicotinamide [28, 43–45]. To our knowledge no such extensive study was performed in hyperlipidemia to demonstrate dose dependant effect *invivo* in combinational therapies of currently used antioxidants. Our study revealed to be parallel with previous data available on other disease conditions confirming the hypothesis of enhancing efficacy of concurrently administered natural antioxidants. Synergism at low combination doses amplifies the insight in reducing the chances of antioxidants to become pro-oxidant. The reduction in LDL/HDL ratio and AI validate the positive outcome of multi antioxidant therapies on lipid profile and blood antioxidants in hyperlipidemic condition [39, 46–48].

The lipophilic antioxidant α-tocopherol, a major antioxidant in LDL prevents peroxidation by getting oxidized by free radicals and administering it alone especially in chronic illnesses will increase cardiovascular risk rather than decreasing it. Administration with exogenous antioxidants was found helpful in α-tocopherol regeneration along with sparing other blood antioxidants from depletion [32, 49]. Synergism was observed using plant phenolic compounds with vitamin C in enhancing LDL resistance towards oxidation [50]. Similarly in our results synergistic effects were observed by combining phenolic compounds with niacin confirmed by decelerated lipid peroxidation. It is reported that use of single antioxidant cannot be able to take the place of combination antioxidant therapy, since giving a single drug in mega dose may behave like prooxidant as observed in case of ascorbic acid [51], curcumin [39] and other natural antioxidants thus may become toxic rather that beneficial.

Similarly the antioxidant effect of catechin, hesperidin, ferulic acid and quercetin were observed at low doses individually up to certain levels but became pro-oxidant as the dose increased above that threshold level, while combinations presented enhanced effects at various doses [40]. This effect is due to the preventive behavior for one another. The same proposal was focused in current research to reduce the chances of single drug dose bulking, system saturation and negative effects. A study on oligomeric proanthocyanidins (OPC) reported dose dependent results up to certain doses [52]. Similarly in another study poor antihypertensive results were obtained in high doses [53], may be due to pro-oxidant effect [54] reducing its own activity. A parallel study on pterostilbene and quercetin revealed significant results in reducing lipid levels at lower doses but at higher doses the effect was not as predicted indicating the ceiling effect [55]. Niacin efficacy in combination with conventional drug therapies and natural antioxidants are already reported of providing synergistic effects on blood lipid profile and improving cardiovascular health in clinical and preclinical studies [56, 57]. It acts dose dependently up to large doses but, the chances of adverse effects like hepatotoxicity [58] and hyperhomocyteineaemia [59] are also reported. However, if combined in low doses with other drugs the adverse events can be minimized along with maximized beneficial outcome. Our proposed therapies were found parallel with several other studies that utilized natural antioxidants in various cardiovascular related conditions [5, 30, 35, 57–60].

Key findings of current study is that combination therapies of natural antioxidants can provide synergistic and additive support for normalizing lipid profile and blood antioxidants in chronic pathological conditions if used below ceiling line. Assessing the serum levels of bio-compounds, the elevated levels of cystine, cysteine, homocysteine, all-*trans* retinoic acid and malondialdehyde validated their involvement in development of hyperlipidemia associated oxidative stress. We developed a dose rationale of selected antioxidants in combination for future use in human equivalent doses in which their efficacies can be evaluated in uncontrolled dietary habits. This will help in developing an efficacy based correlation between preclinical and clinical outcome in preventing atherosclerosis, comparable to conventional drug therapies with negligible side effects.

## Conclusion

In present study hypolipidemic and endogenous antioxidant sparing effects of natural antioxidants were investigated in cholesterol fed rabbits. Results supported the assumption of synergism of natural/dietary antioxidants in combination therapies in chronic ailments. Low dose blend therapies were more effective than individual bulk doses as depicted. We recommend antioxidants in combination dose rationale for achieving maximum benefits in long term therapies.

## Methods

### Chemicals, drugs and reagents

Pure cholesterol (Avonchem UK) (≥99 % pure). Cholesterol kit; TC, TG and HDL (Human diagnostics GmbH Germany). Oligomeric proanthocyanidins (OPC) (Shaanxi Run-Time Bio-Technology Development Co., Ltd), pterostilbene (PT) (Shanghai Korey Pharm Co., Ltd) (≥99 % pure) and niacin (NA) (Scharlau Chemie SA) (≥98 % pure).

### Equipments

HPLC system (Perkin Elmer, Norwalk, USA) linked with a DECADE II Electrochemical Detector (Antec Leyden, Netherland), HPLC column Discovery HS C18 RP chromatographic column (250 mm × 4.6 mm, 5 μm; Bellefonte, USA). Lambda-25 UV/Visible spectrophotometer (Perkin Elmer). Centrifuge machine (Centurion scientific Ltd) and Incubator (Incucell Med Center GmbH Germany) were used in the analysis.

### Hyperlipidemic model development and interventional protocol

Male Albino rabbits (1.2–1.6 kg body weight) were used in the experiment. The experimental protocol for our study was approved by ethical committee of Department of Pharmacy University of Peshawar under reference number: 04/EC-15/Pharm. After 7 days of acclimatization in animal house and bio assay center, hyperlipidemia was developed according to the previous reported protocol [61]. Animals were administered 400 mg/kg cholesterol suspension in 3 ml sunflower oil along with normal diet (protein, fats and fibers each up to 12 %, carbohydrates 65 % and minerals 2–5 %) for about 2 weeks and subsequent analysis of serum samples. Animal having total cholesterol level raised up to 200–250 mg/dl were selected and divided into several groups of *n* = 6. Three groups were treated with OPC, PT and NA individually in 100 mg/kg doses along with cholesterol doses. In 09 groups, low dose combinatorial blends of all the three drugs in 30:70, 50:50 and 70:30 ratios were administered and most effective combination in each two drug combinations was further incorporated in three drug combinations (Table 2). These blends were applied to determine possible extent of synergism of low dose therapies in comparison with individual high dose (100 mg/kg) drug therapy. Atorvastatin (1 mg/kg) was used as standard in group III [62], group I was kept at baseline (saline) and group II on cholesterol only. Duration of therapy was 3 months; the animals were allowed free access to food and water ad libitum. At the end of therapy blood samples were collected in sufficient volumes for lipid profile and chromatographic analysis.

### Laboratory analysis

Plasma lipid profiling was performed on commercial kits. Base line levels were recorded and samples were taken at every 2 weeks interval after overnight fasting to evaluate the effect of therapies on blood lipid levels sequentially. Total cholesterol (TC), teiglyceride (TG) and high density lipoproteins (HDL-C) were calculated enzymatically, while low density lipoproteins (LDL-C), very low density lipoproteins (VLDL-C) and atherogenic index (AI) were calculated according to the following formulae [63–65].

1 $$ \mathrm{L}\mathrm{D}\mathrm{L}-\mathrm{C}=\mathrm{T}\mathrm{C}-\mathrm{H}\mathrm{D}\mathrm{L}-\frac{\mathrm{TG}}{5} $$

2 $$ \mathrm{VLDL}\hbox{-} \mathrm{C}=\frac{\mathrm{TG}}{2.2} $$

3 $$ \mathrm{AI}=\mathrm{L}\mathrm{o}{\mathrm{g}}_{\left(\mathrm{T}\mathrm{G}/\mathrm{H}\mathrm{D}\mathrm{L}\hbox{-} \mathrm{C}\right)} $$

Serum levels of water soluble thiol antioxidants; glutathione (GSH) its oxidized form (GSSG), GSH/GSSG ratio, cystine, cysteine (cys), Homocysteine (Hcy), methionine (meth), N-acetylcysteine (NAC) long with ascorbic acid (AA) and lipid peroxidation were determined simultaneously at the end of study using RP-HPLC- electro chemical detection (ECD) method [31]. Fat soluble antioxidants; α-tocopherol (α-TOH) and all-*trans* retinol (ATR) were determined simultaneously using HPLC-UV [32].

### Statistical analysis

Data analysis was performed by applying student’s *t* test for pair analysis and one way analysis of variance (ANOVA) followed by Dunnett’s test for comparison of lipid lowering effect in all treatment groups with respect to the disease control (with no treatment). Data is presented in mean ± standard error of mean (SEM) at level of significance *p <0.05 %*.

## Abbreviations

AA: Ascorbic acid
AI: Atherogenic index
ANOVA: One way analysis of variance
ATR: All-*trans* retinol
CYP: Cytochrome
Cys: Cysteine
ECD: Electro chemical detection
FFA: Free fatty acids
GSH: Glutathione
GSSG: Glutathione disulfide
Hcy: Homocysteine
HDL: High density lipoproteins
LDL: Low density lipoproteins
Meth: Methionine
NA: Niacin
NAC: N-acetylcysteine
OPC: Oligomeric proanthocyanidins
PPAR: Peroxisome proliferator-activated receptor
PT: Pterostilbene
PUFA: Poly unsaturated fatty acids
ROS: Reactive oxygen species
TG: Triglyceride
VLDL: Very low density lipoproteins
α-TOH: α-tocopherol
